# Association between ERCC2 Lys751Gln, Asp312Asn, and Arg156Arg polymorphisms and gynecological cancer susceptibility: a meta-analysis

**DOI:** 10.3389/fonc.2025.1461015

**Published:** 2025-07-24

**Authors:** Fen Chen, Jiayang Yu, Chun-Guang Wang

**Affiliations:** Department of Oncology, Yongchuan Hospital of Chongqing Medical University, Chongqing, China

**Keywords:** gynecological neoplasms, ovarian cancer, cervical cancer, endometrial cancer, polymorphism, meta-analysis, ERCC2

## Abstract

**Background:**

Gynecological tumors are diseases that pose serious threats to women’s health. Cervical, endometrial, and ovarian cancers are the most common gynecologic tumors. Excision repair cross-complementation group 2 (ERCC2) plays a critical role in nucleotide excision repair. Polymorphisms in ERCC2 can influence DNA damage repair mechanisms, potentially increasing susceptibility to tumors. However, several studies have investigated the association between ERCC2 polymorphisms and the risk of gynecological tumors, but the results have been inconsistent. Therefore, we performed this meta-analysis to estimate these associations more precisely.

**Object:**

In this paper, we summarized a larger sample for meta-analysis to explored the relationship between the polymorphisms of the ERCC2 Lys751Gln, Asp312Asn, and Arg156Arg and gynecological tumors.

**Methods:**

We conducted a systematic search for relevant case-control studies in PubMed, the Cochrane Library, Embase, and the Web of Science databases, covering studies up to October 2024. The odds ratio (OR) and its 95% confidence interval (CI) were calculated using Stata 17 software.

**Results:**

Finally, a total of 19 studies (9433 cases and 13144 controls) were included. 17 studies (3742 cases and 5591 controls) were conducted on the Lys751Gln polymorphism. Additionally, 9 studies(2,170 cases and 3,582 controls)were available for the Asp312Asn polymorphism, while 8 studies (3,521 cases and 3,971 controls)were included for the Arg156Arg polymorphism. Of these, 16 focused on ovarian cancer, 8 on cervical cancer, and 10 on endometrial cancer. The ERCC2 Lys751Gln polymorphism was found to increase the risk of gynecologic neoplasms(C vs A:OR 1.33, 95% CI 1.06-1.66;CC+CA vs AA:OR 1.33, 95% CI 1.11-1.59). Subgroup analysis by cancer type indicated an association of the Lys751Gln polymorphism with the development of ovarian cancer (CC+CA vs AA:OR 1.39, 95% CI 1.04-1.86), while no significant correlation was observed with cervical and endometrial cancers. Further subgroup analyses revealed that the Lys751Gln polymorphism increased the risk of gynecologic neoplasms in Caucasian and African populations, as well as in hospital-based studies. In contrast, the ERCC2 Asp312Asn polymorphism did not elevate the risk of gynecologic neoplasms, and the recessive gene variant was even protective against cervical cancer (AA vs GA+GG : OR 0.53, 95%CI 0.34-0.83, P=0.005). Additionally, this study did not find an association between the Arg156Arg polymorphism and susceptibility to gynecologic tumors.

**Conclusion:**

The ERCC2 Lys751Gln polymorphism is associated with an increased risk of gynecological tumors, particularly ovarian cancer. However, the Asp312Asn and Arg156Arg polymorphisms do not appear to elevate susceptibility to gynecological tumors. Even the recessive gene model of Asp312Asn polymorphism may have a protective effect on cervical cancer.

## Introduction

Gynecological cancer significantly impact women’s health worldwide, with cervical, endometrial, and ovarian cancers being the most common types. In worldwide, cervical, endometrial and ovarian cancer are the fourth, sixth and eighth most common cancers among women, respectively. The number of new cases in 2022 was approximately 661,000, 420,000, and 324,000, respectively. In terms of mortality, cervical and ovarian cancers ranked fourth and eighth among worldwide female, accounting for approximately 348,000 and 207,000 deaths, respectively (1). Genetic factors play a crucial role in the development of gynecological tumors, particularly ovarian and endometrial cancers. A report of familial clustering of cervical cancer suggest that genetic factors may contribute to the occurrence of cervical cancer (2). The survival rate of cervical cancer exhibits significant regional variation. In countries with a high Human Development Index (HDI), the 5-year survival rate of cervical cancer is 60-70%, while in countries with a low HDI, it drops to less than 20% (3). Endometrial cancer is the second most common gynecological cancer after cervical cancer. According to the 2023 Cancer Statistics Report, the incidence of endometrial cancer continues to rise (4). Estrogen plays a pivotal role in the development of endometrial cancer. Estrogen produces DNA bulk adducts and oxidative base damage. Base excision repair (BER) and nucleotide excision repair (NER) systems are important pathways to remove these lesions (5, 6).Ovarian cancer has a poor prognosis, which is one of the gynecological malignancies with a relatively high mortality rate. Age, reproductive history, family history, and lifestyle are high-risk factors for ovarian cancer (7). Family history is one of the most significant risk factors for ovarian cancer. Mutations in BRCA1 and BRCA2 are strongly associated with hereditary ovarian cancer (8).

The DNA repair system in the human body can repair damage to maintain the stability of the genome. The major DNA repair pathways include base excision repair (BER), nucleotide excision repair(NER), mismatch repair(MMR), homologous recombination (HR), and non-homologous end joining (NHEJ) (9). The development of tumors is influenced by both genetic factors and environmental conditions. The impaired DNA damage repair playing a key role in promoting tumor formation. Single nucleotide polymorphism (SNP) is one of the most common forms of human genetic variation. It affects the repair ability of damaged DNA by regulating gene expression or altering the function of gene products, thereby increasing the susceptibility to cancer (10).

NER pathway is one of the most important DNA repair system in humans, removing damage caused by physical and chemical carcinogens. This pathway repairs UV-induced photoproducts, bulky DNA adducts, chemotherapy-induced intrastrand cross-links, and other helix-distorting lesions (11). NER works through two main mechanisms: global genome repair (GG-NER)and transcription-coupled repair (TC-NER) (12). It is essential for fixing many types of DNA damage. If NER fails, DNA damage builds up, which can cause genomic instability and increase cancer risk.

Excision repair cross Complementation Group 2(ERCC2), also known as Xeroderma Pigmentosum Complementation Group D (XPD), palys an important role in DNA repair through the nucleotide excision repair pathway. ERCC2 gene is located on chromosome 19q13.3 and comprises 23 exons covering approximately 54,000 base pairs. In the coding region of ERCC2, several common polymorphisms have been identified, including Lys751Gln (rs13181), Asp312Asn (rs1799793), and Arg156Arg (rs238406). The Lys751Gln polymorphism involves the substitution of lysine (Lys) with glutamine (Gln) at position 751 within exon 23. The Asp312Asn polymorphism refers to the replacement of aspartic acid (Asp) at position 312 in exon 10 with asparagine (Asn). The Arg156Arg polymorphism entails a base change from cytosine to adenine at position 156 in exon 6; however, this alteration does not result in an amino acid change and continues to encode arginine (Arg) (13). ERCC2 protein is a highly conserved ATP-dependent DNA helicase, which is one of the proteins that constitute the Transcription Factor IIH (TFIIH) complex. The TFIIH complex mediates the initiation of transcription, participates in the separation of the double helix, and recruits downstream repair factors during the NER process. The TFIIH complex consists of a core of seven subunits (composed of XPB, XPD, p52, p8, p62, p34 and p44) and CAK(composed of CDK7, Cyclin H and MAT1) (11). Within the TFIIH complex, the XPB and XPD proteins are responsible for untwisting the DNA double helix structure and separating the DNA strands during the DNA repair process (14).

The ERCC2 gene exhibits high levels of genetic polymorphism and has been associated with increased susceptibility to various types of cancer, including lung cancer (15), hepatocellular carcinoma (16), and malignant melanoma (17). However, the relationship between ERCC2 gene polymorphisms and susceptibility to gynecological tumors remains unclear. To address this gap, we performed a comprehensive literature review incorporating multiple relevant clinical studies and applied meta-analysis methods to systematically evaluate the correlations between ERCC2 Lys751Gln, Asp312Asn, and Arg156Arg SNP and the risk of cervical cancer, endometrial cancer, and ovarian cancer.

## Materials and methods

### Literature search strategy

A computerized search was conducted in databases including PubMed, the Cochrane Library, Embase, Web of Science for relevant literature on the correlation between nucleotide excision repair gene ERCC2 polymorphisms (Lys751Gln, Asp312Asn, R156R) and cervical, endometrial, and ovarian cancers. The search was from the establishment of the library to September 2023. The search terms used included “Genital Neoplasms”, ”ovarian neoplasms”, ”Cervical Neoplasms”, ”Endometrial Neoplasms”, ”polymorphism”, ”variant”, ”genotype”, ”SNP”, ”polymorphisms”, ”Single Nucleotide Polymorphism”, ”ERCC2 protein” , ”XPD”, ”Xeroderma Pigmentosum Group D”, ”excision repair cross-complementing rodent repair deficiency 2”, ”rs13181”, ”rs238406”, ”rs1799793”.

### Inclusion and exclusion criteria

#### Inclusion criteria

(1) case-control studies on gynecologic patients and non-cancer populations; (2) The studies that assessed the associations between ERCC2 polymorphisms (Lys751Gln, Asp312Asn, and Arg156Arg) and the risk of gynecologic cancer; (3) Sufficient genotype data in both the case and control groups to calculate the odds ratios (ORs) with 95% confidence intervals (95% CIs).

#### Exclusion criteria

(1) Non-case-control studies. (2) Review articles, meta-analyses, case reports, animal experiments. (3) Overlapping studies. (4) Inaccessible full-text articles.(5)Studies based on family.

### Literature screening, data extraction and quality assessment

Two researchers independently screened the literature to select eligible studies and extracted relevant data, including: first author, publication year, ethnicity, cancer type, single nucleotide polymorphism, control type, genotyping method, source of controls. The third researcher CG-W was asked to discuss the disagreement in order to reach a final conclusion. The quality of the included literature was evaluated using the Newcastle-Ottawa (NOS) Scale.

### Statistical analysis

The three genetic models: allele model (A vs a), dominant model (AA + Aa vs aa), and recessive model (AA vs Aa + aa) are used to assess the association between ERCC2 polymorphisms and the risk of cervical cancer, endometrial cancer, and ovarian cancer. Subgroup analyses were conducted based on cancer type, ethnicity, source of population, and Hardy-Weinberg equilibrium (HWE). Data analysis was performed using Stata 17 software. Pooled odds ratios (ORs) with 95% confidence intervals (95% CIs) were calculated as the effect size. A statistically significant association was considered when P < 0.05. Heterogeneity was evaluated using the Q test and I^2^ test. If *P_h_* >0.1 or *I^2^* < 50%, a fixed-effects model was used; otherwise, a random-effects model was applied. Sensitivity analysis evaluated result stability by iteratively excluding one study at a time and comparing outcomes. Publication bias was initially assessed by funnel plot and confirmed using Egger’s test (P < 0.05 indicating bias).

## Results

### Characteristics of included studies

**Figure 1:**
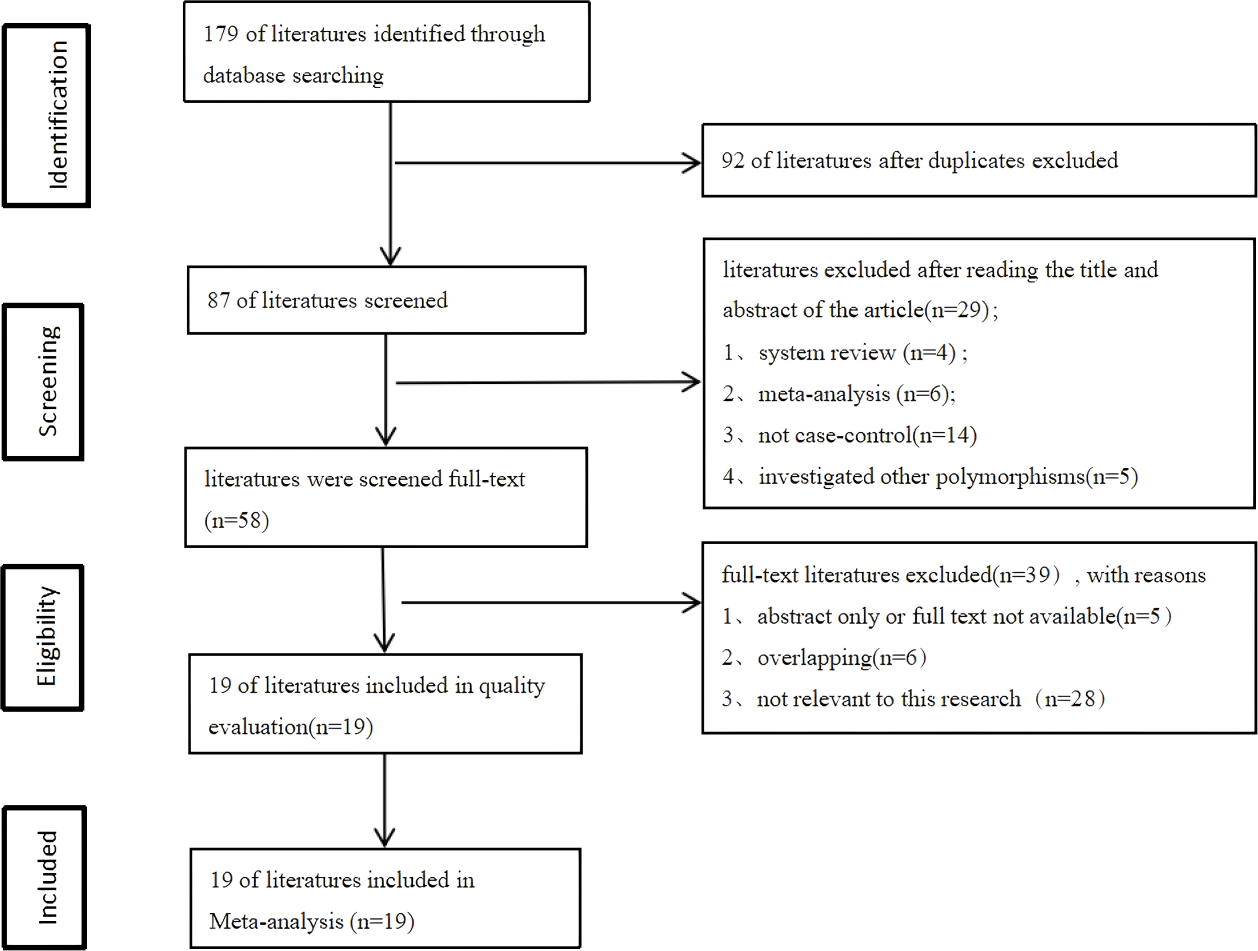
Literature screening process.

After searching the databases, a total of 179 relevant articles were retrieved (50 from PubMed, 2 from the Cochrane Library, 76 from Embase, and 51 from Web of Science). Following inclusion and exclusion criteria, a total of included 19 articles (6, 18–35) including 34 studies were finally included. All 19 included articles (9433 cases and 13144 controls)were case-control studies, and the quality of included studies was assessed using the Newcastle-Ottawa Scale (NOS). The NOS scores of all included studies ranged from 5 to 7 scores. The process of literature search and screening is shown in **Figure 1**, and the basic information and quality evaluation results of the included studies are shown in **Tables 1**–**3**. In these studies, 17 were conducted on Lys751Gln polymorphism, 9 on Asp312Asn polymorphism, 8 on Arg156Arg polymorphism, respectively. In all the included studies, 16 were about OC and 8 were about CC and 10 were about EC.

**Table 1:** Basic information about the ERCCR2 Lys751Gln polymorphism.

Study ID	Year	Cancer type	SOC	Ethnicity	Method	Case			Control			HWE	NOS score
						AA	AC	CC	AA	AC	CC		
Costa (21)	2007	OC	HB	Caucasian	PCR–RFLP	55	49	22	95	95	12	Y	7
Bernard (19)	2008	OC	HB	Caucasian	TaqMan	1	31	19	119	446	430	N	5
Jakubowska (25)	2009	OC	HB	Caucasian	PCR–RFLP	58	65	22	100	123	57	Y	6
Khokhrin (26)	2012	OC	HB	Caucasian	PCR–RFLP	28	54	22	109	143	46	Y	6
Mohamed (28)	2013	OC	HB	African	PCR–RFLP	32	54	14	55	35	10	Y	7
Monteiro (29)	2014	OC	HB	Caucasian	PCR-RFLP	33	36	1	37	30	3	N	6
Michalska2 (27)	2015	OC	HB	Caucasian	PCR-RFLP	62	64	304	96	240	94	N	7
Zhao (35)	2018	OC	HB	Asian	Taqman & ABI Prism	74	15	0	296	59	1	Y	7
He (24)	2008	CC	HB	Asian	PCR	165	33	2	164	31	5	Y	5
Zhang (34)	2011	CC	HB	Asian	PCR	68	12	0	148	27	1	Y	7
Bajpai (18)	2016	CC	HB	Asian	PCR-RFLP	27	24	14	52	9	7	N	7
Datkhik (22)	2022	CC	HB	Asian	PCR-RFLP	178	197	25	187	181	32	N	6
Weiss (33)	2005	EC	PB	Caucasian	PCR-RFLP	142	181	48	159	197	64	Y	7
Doherty (23)	2011	EC	PB	Caucasian	SNPlex/TaqMan/ABI/RFLP/fragment analyses	269	347	87	282	333	99	Y	7
Cincin (20)	2012	EC	HB	Caucasian	PCR–RFLP	20	35	49	61	22	75	N	5
Sobczuk (32)	2012	EC	HB	Caucasian	PCR-RFLP	30	36	28	38	64	12	Y	6
Smolarz (31)	2018	EC	HB	Caucasian	PCR-RFLP	152	154	304	186	240	184	N	5

OC, Ovarian Cancer; CC, Cervical Cancer; EC, Endometrial Cancer; SOC, source of controls; PB, population-based controls; HB, hospital-based controls; HWE Hardy–Weinberg equilibrium, Y in agreement with HWE, N not in agreement with HWE; NOS, Newcastle-Ottawa Scale.

**Table 2:** Basic information about the ERCCR2 Asp312Asn polymorphism.

Study ID	Year	Cancer Type	SOC	Ethnicity	Method	Case			Control			HWE	NOS score
						GG	GA	AA	GG	GA	AA		
Costa (21)	2007	OC	HB	Caucasian	PCR–RFLP	62	33	19	109	75	15	N	7
Bernard (19)	2008	OC	HB	Caucasian	Taqman	21	28	2	458	418	118	Y	5
Jakubowska (25)	2009	OC	HB	Caucasian	PCR–RFLP	59	59	26	102	129	49	Y	6
Khokhrin (26)	2012	OC	HB	Caucasian	PCR–RFLP	34	50	20	106	145	47	Y	6
Monteiro (29)	2014	OC	HB	Caucasian	PCR-RFLP	8	29	33	9	20	41	Y	6
He (24)	2008	CC	HB	Asian	PCR	90	93	17	79	89	32	Y	5
Datkhile (22)	2022	CC	HB	Asian	PCR-RFLP	345	39	16	198	175	27	N	6
Weiss (33)	2005	EC	PB	Caucasian	PCR-RFLP	152	173	46	186	176	58	Y	7
Doherty (23)	2011	EC	PB	Caucasian	SNPlex/TaqMan/ABI/RFLP/fragment analyses	291	350	75	318	313	90	Y	7

OC, Ovarian Cancer; CC, Cervical Cancer; EC, Endometrial Cancer; SOC, source of controls; PB, population-based controls; HB, hospital-based controls; HWE Hardy–Weinberg equilibrium, Y in agreement with HWE, N not in agreement with HWE; NOS, Newcastle-Ottawa Scale.

**Table 3:** Basic information about the ERCCR2 Arg156Arg polymorphism.

Study ID	Year	Cancer Type	SOC	Ethnicity	Method	Case			Control			HWE	NOS score
						CC	CA	AA	CC	CA	AA		
Costa (21)	2007	OC	HB	Caucasian	PCR–RFLP	36	61	21	38	109	40	Y	7
Romanowicz (30)	2016	OC	HB	Caucasian	PCR-RFLP	76	135	189	122	186	92	N	6
Zhao (35)	2018	OC	HB	Asian	Taqman & ABI Prism	13	44	32	95	168	93	Y	7
Zhang (34)	2011	CC	HB	Asian	PCR	29	36	15	55	83	36	Y	7
Datkhik (22)	2022	CC	HB	Asian	PCR-RFLP	126	212	62	145	185	70	Y	6
Weiss (33)	2005	EC	PB	Caucasian	PCR-RFLP	117	188	66	137	207	76	Y	7
Doherty (23)	2011	EC	PB	Caucasian	SNPlex/TaqMan/ABI/RFLP/fragment analyses	207	367	129	219	361	134	Y	7
Michalska (6)	2015	EC	HB	Caucasian	PCR-RFLP	136	144	1080	264	840	216	N	6

OC, Ovarian Cancer; CC, Cervical Cancer; EC, Endometrial Cancer; SOC, source of controls; PB, population-based controls; HB, hospital-based controls; HWE Hardy–Weinberg equilibrium, Y in agreement with HWE, N not in agreement with HWE; NOS, Newcastle-Ottawa Scale.

### Meta-analysis data

**Figure 2:**
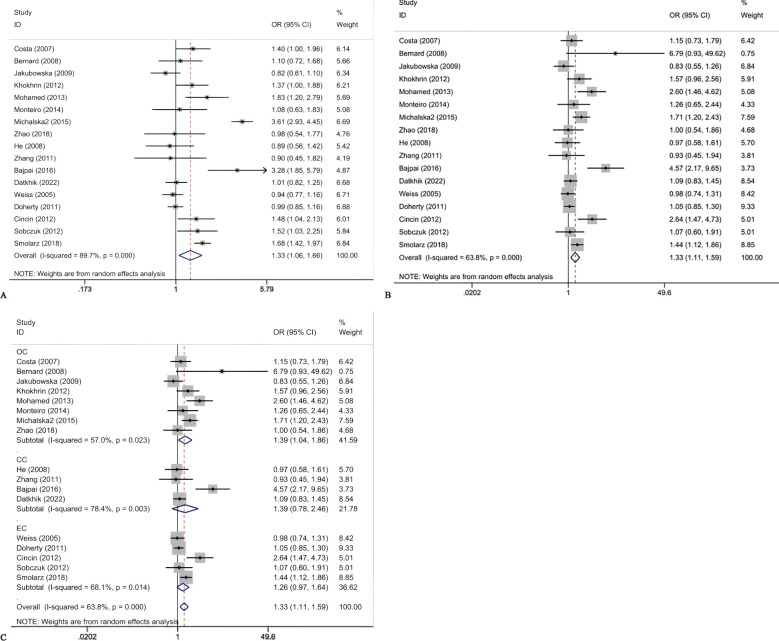
Forest plots for ERCCR2 Lys751Gln C vs A **(A)**; ERCCR2 Lys751Gln CC + AC vs AA **(B)**; ERCCR2 Lys751Gln CC + AC vs AA in cancer type subgroups **(C)**.

The association between ERCC2 Lys751Gln polymorphism and the risk of CC, EC, and OC is as follows (**Table 4**). This study found that there is an association between ERCC2 gene Lys751Gln polymorphism and increased risk of gynecological tumors, particularly increasing the risk of ovarian cancer (**Figure 2**). The meta-analysis suggested C vs A: OR:1.33(95% CI 1.06-1.66), CC+CA vs AA OR 1.33(95% CI:1.11-1.59). Analysis based on cancer types suggested that Lys751Gln polymorphism is associated with ovarian cancer (CC+CA vs AA:OR 1.39, 95% CI 1.04-1.86), but not significantly correlated with cervical cancer or endometrial cancer. Subgroup analysis by ethnic groups revealed that in Caucasian and African populations, Lys751Gln polymorphism increases the risk of gynecological tumors. In the allele genetic model C vs A, the OR values for Caucasian and African populations were 1.34 (95% CI 1.01-1.78) and 1.83 (95% CI 1.20-2.79), respectively; in the dominant genetic model, the OR values for Caucasian and African populations were 1.28(95% CI 1.06-1.55)and 2.60(95%CI 1.46-4.62), respectively. Subgroup analysis by population source revealed that in hospital populations, Lys751Gln polymorphism is associated with the risk of gynecological tumors (C vs A:OR:1.39, 95% CI 1.08-1.79;CC+CA vs AA:OR 1.41, 95% CI 1.15-1.74). Subgroup analyses according to HWE found that allele models and dominant gene models were associated with the development of gynecological tumors in those who did not comply with the HWE (C vs A:OR 1.66, 95CI 1.11-2.49;CC+ AC vs AA:OR 1.77, 95CI 1.27-2.45).

**Table 4:** Subgroup analysis of ERCCR2 Lys751Gln.

Comparison	Study groups	Study groups	Studies	OR (95% CI)	*P*	Heterogeneity		Effect model
						*P_h_*	*I^2^* (%)	
Allele C vs A Dominant CC + AC vs AA Recessive CC vs AA+AC	Overall Cancer Type SOC HWE Ethnicity Overall Cancer Type SOC HWE Ethnicity Overall Cancer Type SOC HWE Ethnicity	OC CC EC HB PB Y N Caucasian Asian African OC CC EC HB PB Y N Caucasian Asian African OC CC EC HB PB Y N Caucasian Asian African	17 8 4 5 15 2 10 7 11 5 1 17 8 4 5 15 2 10 7 11 5 1 17 8 4 5 15 2 10 7 11 5 1	1.33 (1.06-1.66) 1.38 (0.89-2.14) 1.26 (0.75-2.12) 1.27 (0.97-1.66) 1.39 (1.08-1.79) 0.98 (0.86-1.10) 1.12 (0.96-1.31) 1.66 (1.11-2.49) 1.34 (1.01-1.78) 1.20 (0.79-1.82) 1.83 (1.20-2.79) 1.33 (1.11-1.59) 1.39 (1.04-1.86) 1.39 (0.78-2.46) 1.26 (0.97-1.64) 1.41 (1.15-1.74) 1.03 (0.87-1.22) 1.11 (0.94-1.31) 1.77 (1.27-2.45) 1.28 (1.06-1.55) 1.29 (0.82-2.03) 2.60 (1.46-4.62) 1.39 (0.87-2.21) 1.55 (0.61-3.97) 0.98 (0.46-2.10) 1.35 (0.81-2.24) 1.50 (0.89-2.53) 0.86 (0.67-1.10) 1.26 (0.84-1.87) 1.54 (0.69-3.42) 1.52 (0.86-2.67) 0.99 (0.52-1.86) 1.47 (0.62-3.47)	0.013 0.147 0.385 0.085 0.009 0.696 0.162 0.014 0.042 0.397 0.005 0.002 0.027 0.262 0.086 0.001 0.757 0.230 0.001 0.011 0.272 0.001 0.170 0.359 0.953 0.249 0.125 0.222 0.259 0.290 0.148 0.968 0.386	0.000 0.000 0.001 0.000 0.000 0.688 0.014 0.000 0.000 0.003 - 0.000 0.023 0.003 0.014 0.001 0.704 0.140 0.003 0.009 0.007 - 0.000 0.000 0.164 0.000 0.000 0.818 0.001 0.000 0.000 0.271 -	89.7 92.0 81.0 86.6 88.3 0.0 56.5 92.9 92.4 74.9 - 63.8 57.0 78.4 68.1 62.5 0.0 33.5 70.3 57.2 71.8 - 91.2 93.4 41.3 88.5 90.2 0.0 67.9 94.7 94.1 22.5 -	R R R R R F R R R R - R R R R R F F R R R - R R F R R F R R R F -

OC, Ovarian Cancer; CC, Cervical Cancer; EC, Endometrial Cancer; SOC, source of controls; PB, population-based controls; HB, hospital-based controls; HWE, Hardy–Weinberg equilibrium, Y in agreement with HWE, N not in agreement with HWE; R, random effect model; F, fixed effect model.

**Figure 3:**
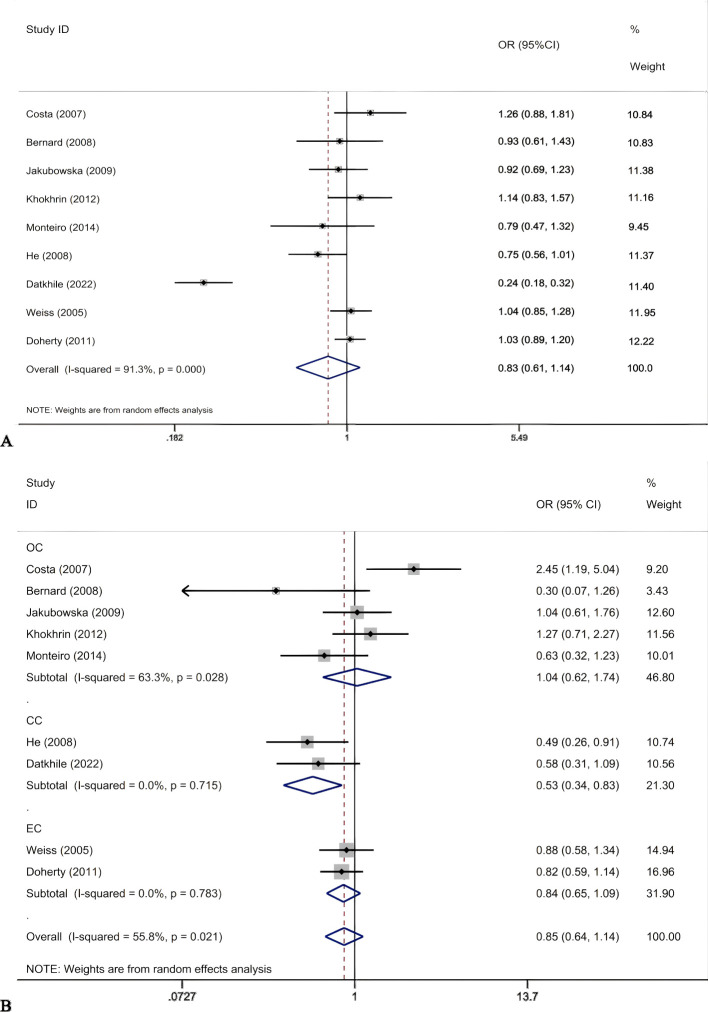
Forest plots for ERCCR2 Asp312Asn A vs G **(A)**; ERCC2Asp312Asn AA vs GG+GA in cancer type subgroups **(B)**.

The results of meta-analysis of the association between ERCC2 Asp312Asn polymorphisms and gynecological tumors were as follows (**Table 5**). A total of 2170 tumor patients and 3582 healthy controls were included in the 9 studies. The results showed that (**Figure 3**) in the recessive gene model, in the meta-analysis with cancer type subgroups found that recessive genes AA vs GA+GG reduced the risk of cervical cancer (OR 0.53, 95%CI 0.34-0.83, P=0.005). Subgroup analyses by ethnicity suggested that the recessive gene model reduced the risk of gynecologic neoplasms in Asian women (OR 0.53, 95% CI 0.34-0.83, P=0.005). In contrast, the pooled analysis did not suggest a correlation. In the recessive gene model, AA vs GA+GG: OR 0.85, 95%CI 0.64-1.14, P=0.278; in the dominant gene model, AA + GA vs GG: OR 0.83, 95%CI 0.52-1.34, P=0.445; and in the allele model, A vs C:OR = 0.83, 95%CI 0.61-1.14, P=0.253, none of the results were statistically significant. In addition, there was no suggestion of an increased risk of gynecologic cancer in the subgroup analyses (by source of population, HWE). The ERCC2 Asp312Asn polymorphism was not significantly associated with gynecological tumors, and the recessive gene was protective against cervical cancer. In addition, in Asian women, the recessive gene reduces the risk of cervical cancer.

**Table 5:** Subgroup analysis of ERCCR2 Asp312Asn.

Comparison	Study groups	Study groups	Studies	OR (95% CI)	*P*	Heterogeneity		Effect model
*P_h_*	*I^2^* (%)							
Allele A vs G Dominant AA+ GA vs GG Recessive AA vs GA+GG	Overall Cancer Type SOC HWE Ethnicity Overall Cancer Type SOC HWE Ethnicity Overall Cancer Type SOC HWE Ethnicity	OC CC EC HB PB Y N Caucasian Asian OC CC EC HB PB Y N Caucasian Asian OC CC EC HB PB Y N Caucasian Asian	9 5 2 2 7 2 7 2 7 2 9 5 2 2 7 2 7 2 7 2 9 5 2 2 7 2 7 2 7 2	0.83 (0.61-1.14) 1.02 (0.87-1.20) 0.43 (0.14-1.29) 1.04 (0.92-1.17) 0.78 (0.49-1.23) 1.04 (0.92-1.17) 0.98 (0.89-1.08) 0.55 (0.11-2.78) 1.03 (0.93-1.14) 0.43 (0.14-1.29) 0.83 (0.52-1.34) 1.01 (0.81-1.27) 0.35 (0.07-1.74) 1.15 (0.97-1.36) 0.76 (0.39-1.46) 1.15 (0.97-1.36) 1.07 (0.93-1.22) 0.40 (0.06-2.48) 1.10 (0.96-1.26) 0.35 (0.07-1.74) 0.85 (0.64-1.14) 1.04 (0.62-1.74) 0.53 (0.34-0.83) 0.84 (0.65-1.09) 0.84 (0.54-1.31) 0.84 (0.65-1.09) 0.82 (0.64-1.04) 1.18 (0.28-4.87) 0.97 (0.71-1.32) 0.53 (0.34-0.83)	0.253 0.796 0.132 0.572 0.282 0.572 0.703 0.471 0.545 0.132 0.445 0.909 0.200 0.104 0.405 0.104 0.335 0.322 0.168 0.200 0.278 0.892 0.005 0.194 0.443 0.194 0.094 0.823 0.844 0.005	0.000 0.486 0.000 0.942 0.000 0.942 0.448 0.000 0.747 0.000 0.000 0.802 0.000 0.972 0.000 0.972 0.596 0.000 0.879 0.000 0.021 0.028 0.715 0.783 0.006 0.783 0.220 0.003 0.051 0.715	91.3 0.0 96.6 0.0 92.2 0.0 0.0 98.0 0.0 96.6 92.7 0.0 97.3 0.0 92.7 0.0 0.0 97.5 0.0 97.3 55.8 63.3 0.0 0.0 66.7 0.0 27.3 88.6 52.2 0.0	R F R F R F F R F R R F R F R F F R F R R R F F R F F R R F

OC, Ovarian Cancer; CC, Cervical Cancer; EC, Endometrial Cancer; SOC, source of controls; PB, population-based controls; HB, hospital-based controls; HWE, Hardy–Weinberg equilibrium, Y in agreement with HWE, N not in agreement with HWE; R, random effect model; F, fixed effect model.

**Figure 4:**
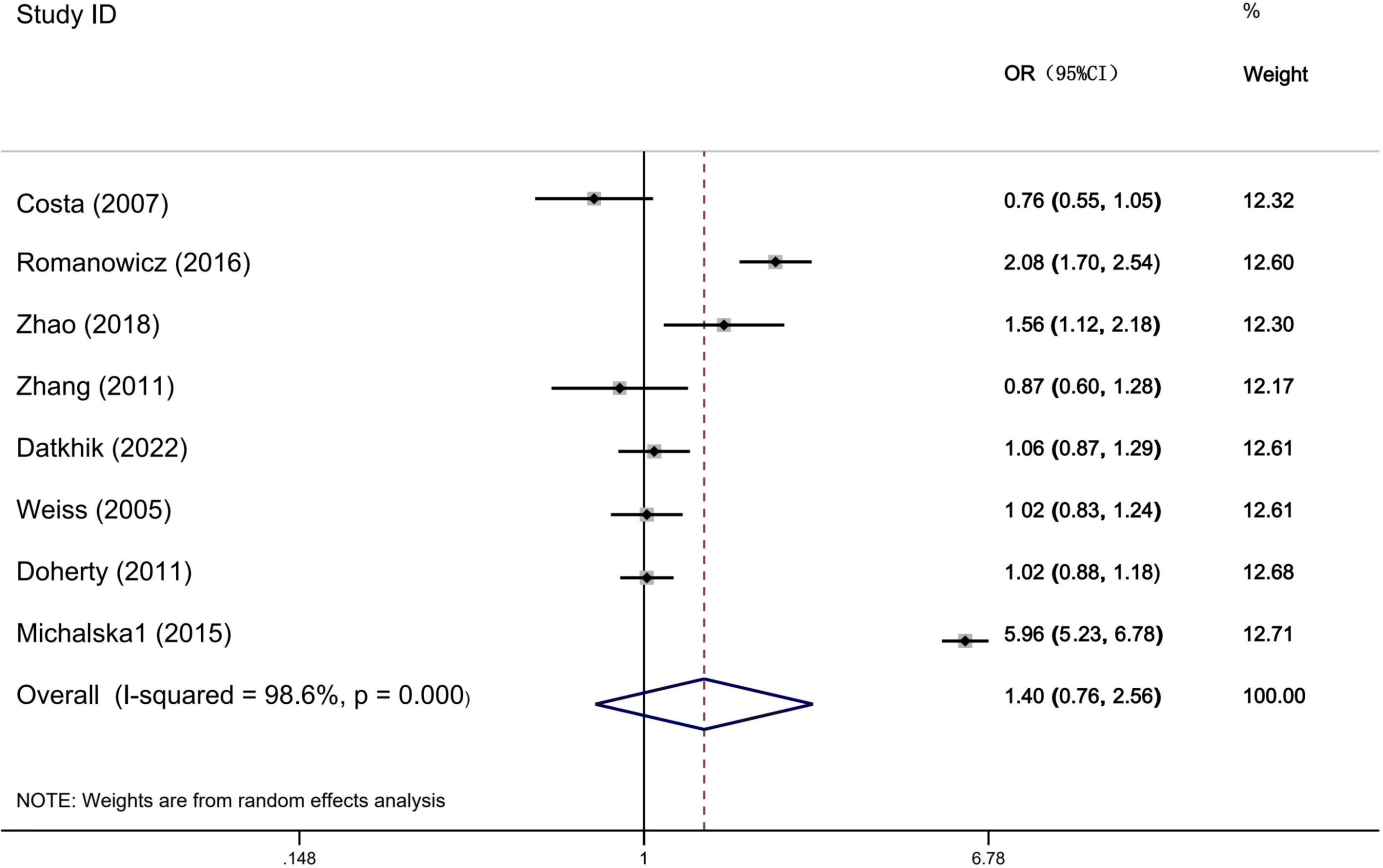
Forest plots for ERCCR2 Arg156Arg A vs C.

**Table 6** shows that the meta-analysis results on the relationship between ERCC2 Arg156Arg polymorphism and common gynecological tumors (**Figure 4**). A total of eight studies were included, including 3521 cases and 3971 controls. Only in the subgroup analysis of HWE, the results indicate that in the population not meeting the law of HWE, allele model A vs C and dominant gene model AA + CA vs CC were associated with the development of gynecological tumors. Additionally, no significant correlation was found between the ERCC2 Arg156Arg polymorphism and common gynecological tumors, including ovarian cancer, cervical cancer, and endometrial cancer (A vs C: OR 1.40, 95% CI 0.76-2.56, P= 0.278; AA + CA vs CC: OR 1.27, 95% CI 0.93-1.72, P =0.127; AA vs CA+CC: OR 1.66, 95% CI 0.57-4.82, P =0.351). Subgroup analysis (source of population, ethnicity) yielded similar results, indicating no statistically significant association between the Arg156Arg polymorphism and gynecological tumors. Therefore, the ERCC2 Arg156Arg (C/A) polymorphism does not increase the risk of ovarian cancer, cervical cancer, or endometrial cancer.

**Table 6:** Subgroup analysis of ERCCR2 Arg156Arg.

Comparison	Study groups	Study groups	Studies	OR (95% CI)	*P*	Heterogeneity		Effect model
						*P_h_*	*I^2^* (%)	
Allele A vs C Dominant AA + CA vs CC Recessive AA vs CA+CC	Overall Cancer Type SOC HWE Ethnicity Overall Cancer Type SOC HWE Ethnicity Overall Cancer Type SOC HWE Ethnicity	OC CC EC HB PB Y N Caucasian Asian OC CC EC HB PB Y N Caucasian Asian OC CC EC HB PB Y N Caucasian Asian	8 3 2 3 6 2 6 2 5 3 8 3 2 3 6 2 6 2 5 3 8 3 2 3 6 2 6 2 5 3	1.40 (0.76-2.56) 1.36 (0.75-2.47) 1.02 (0.85-1.21) 1.83 (0.52-6.44) 1.55 (0.73-3.32) 1.02 (0.90-1.14) 1.02 (0.89-1.18) 3.53 (1.26-9.91) 1.58 (0.68-3.66) 1.13 (0.84-1.51) 1.27 (0.93-1.72) 1.33 (0.61-2.88) 1.07 (0.73-1.58) 1.36 (0.81-2.30) 1.35 (0.91-2.01) 1.06 (0.88-1.27) 1.05 (0.83-1.33) 2.12 (1.77-2.55) 1.26 (0.83-1.91) 1.26 (0.81-1.96) 1.66 (0.57-4.82) 1.61 (0.75-3.45) 0.87 (0.63-1.21) 2.67 (0.29-24.24) 1.99 (0.54-7.25) 0.98 (0.79-1.21) 0.98 (0.84-1.16) 7.72 (1.22-48.90) 2.16 (0.51-9.04) 1.07 (0.71-1.60)	0.278 0.311 0.864 0.344 0.255 0.789 0.731 0.017 0.285 0.408 0.127 0.477 0.716 0.243 0.137 0.550 0.657 0.000 0.277 0.309 0.351 0.221 0.401 0.384 0.299 0.819 0.844 0.030 0.294 0.775	0.000 0.000 0.382 0.000 0.000 0.997 0.072 0.000 0.000 0.057 0.000 0.001 0.191 0.000 0.000 0.964 0.042 0.362 0.000 0.081 0.000 0.000 0.954 0.000 0.000 0.976 0.456 0.000 0.000 0.136	98.6 92.4 0.0 99.5 98.5 0.0 50.5 98.7 99.1 65.0 84.9 86.8 41.4 92.5 85.0 0.0 56.7 0.0 90.1 60.1 98.7 88.2 0.0 99.5 98.6 0.0 0.0 99.0 99.1 49.8	R R F R R F R R R R R R F R R F R F R R R R F R R F F R R F

OC, Ovarian Cancer; CC, Cervical Cancer; EC, Endometrial Cancer; SOC, source of controls; PB, population-based controls; HB, hospital-based controls; HWE, Hardy–Weinberg equilibrium, Y in agreement with HWE, N not in agreement with HWE; R, random effect model; F, fixed effect model.

### Publication bias

**Figure 5:**
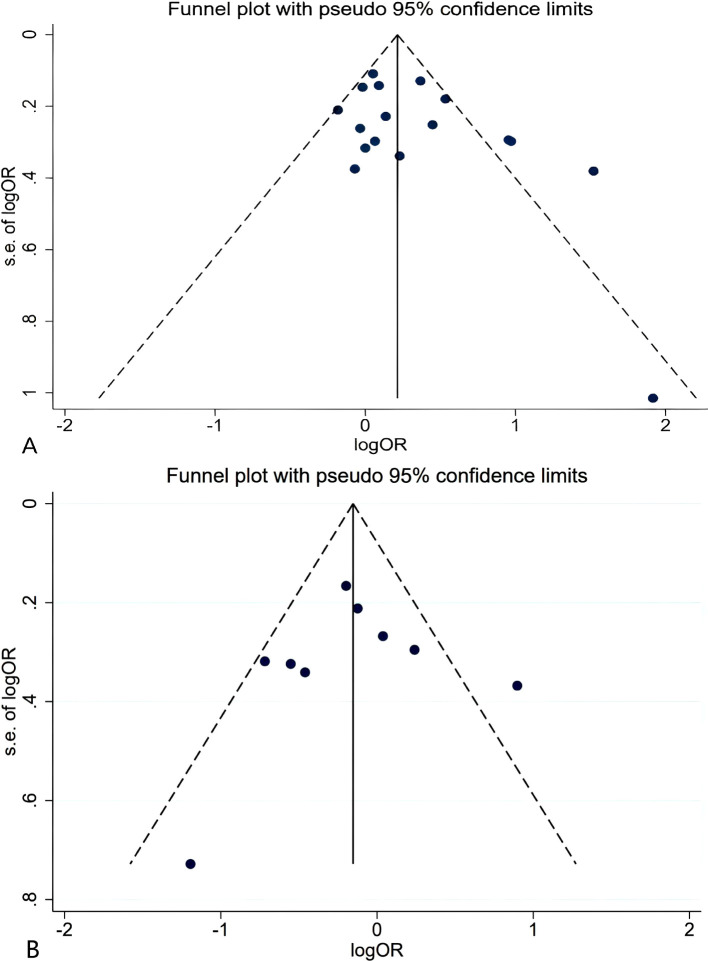
Funnel plot for ERCCR2 Lys751Gln CC+AC vs AA **(A)**; Asp312Asn AA vs GA+GG **(B)**.

We assessed publication bias using a funnel plot, which showed a relatively symmetrical distribution of studies (**Figure 5**). Further evaluation using Egger’s test for publication bias regarding ERCC2 Lys751Gln (C vs A: P=0.993, CC + AC vs AA: P=0.082, CC vs AA+AC: P=0.351), ERCCR2 Asp312Asn (A vs G: P=0.593, AA+ GA vs GG: P=0.825, AA vs GA+GG: P=0.735), and ERCCR2 Arg156Arg (A vs C: P=0.179, AA + CA vs CC: P=0.426, AA vs CA+CC: P=0.058) revealed p-values greater than 0.05 for all comparisons, indicating no statistically significant publication bias (**Table 7**). so it did not suggest the existence of publication bias.

**Table 7:** Publication bias.

ERCC2	Std_Eff	Coef.	Std. Err.	t	P>|t|	[95% CI]	
Lys751Gln C vs A	slope bias	0.2896949 -0.0174784	0.2606204 1.844675	1.11 -0.01	0.284 0.993	-0.2658042 -3.94931	0.8451941 3.914354
Lys751Gln CC+AC vsAA	slope bias	-0.090675 1.656517	0.1805974 0.8880061	-0.50 1.87	0.623 0.082	-0.4756092 -0.2362235	0.2942592 3.549257
Lys751Gln CC vs AA+AC	slope bias	0.8565168 -1.481961	0.3909956 1.540864	2.19 -0.96	0.045 0.351	0.0231294 -4.766235	1.689904 1.802314
Asp312Asn A vs G	slope bias	0.1082004 -1.915482	0.4623377 3.419389	0.23 -0.56	0.822 0.593	-0.9850545 -10.00105	1.201455 6.170088
Asp312Asn AA+ GA vs GG	slope bias	-0.0318854 -0.811002	0.6501087 3.530566	-0.05 -0.23	0.962 0.825	-1.569148 -9.159464	1.505377 7.53746
Asp312Asn AA vs GG+GA	slope bias	-0.0104458 -0.5564856	0.430895 1.58165	-0.02 -0.35	0.981 0.735	-1.029351 -4.296493	1.008459 3.183522
Arg156Arg A vs C	slope bias	1.775862 -12.18975	0.805258 8.003561	2.21 -1.52	0.070 0.179	-0.194533 -31.77376	3.746257 7.394255
Arg156Arg AA + CA vs CC	slope bias	0.6716398 -2.35113	0.4413517 2.750882	1.52 -0.85	0.179 0.426	-0.408309 -9.082297	1.751589 4.380036
Arg156Arg AA vs CA+CC	slope bias	3.608051 -15.55362	1.109055 6.637537	3.25 -2.34	0.017 0.058	0.8942918 -31.79509	6.32181 0.6878458

### Sensitivity analysis

**Figure 6:**
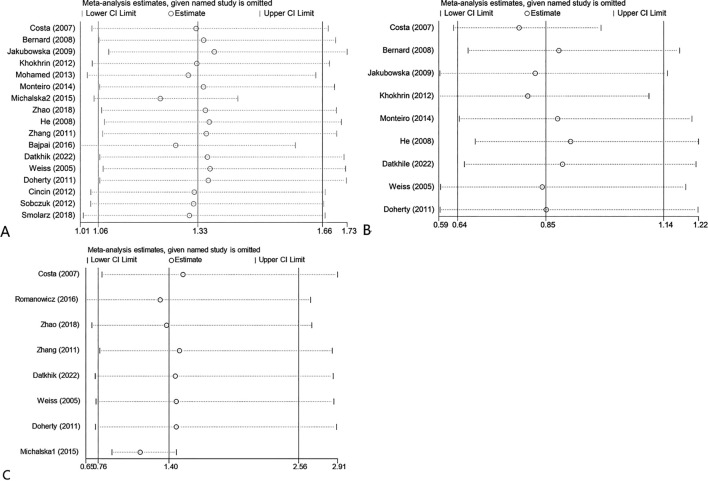
Sensitivity analysis about ERCC2 Lys751Gln C vs A **(A)**; Asp312Asn AA vs GG+GA **(B)**; Arg156Arg A vs C **(C)**.

We conducted sensitivity analyses by excluding each study and comparing the change in the pooled odds ratio (ORs) and their 95% CI. **Figure 6** illustrates that, for the sensitivity analysis of the ERCC2 Lys751Gln, Asp312Asn, and Arg156Arg polymorphisms, there was no significant change in the pooled ORs and 95% CI after removing either study. The results of this study were stable and reliable.

## Discussion

The development of gynecological tumors result from the combined effects of several factors, including genes and the environment. Abnormal DNA damage repair function is related to the development of cancer. In recent years, progress has been made in understanding the pathogenesis and anti-cancer treatment of gynecological tumors. However, the 5-year survival rate for gynecological tumors, particularly ovarian cancer, remains low.

The ERCC2 gene encodes the ERCC2 protein, which plays a crucial role in the NER process. Polymorphisms in the ERCC2 gene may be associated with tumor susceptibility. The Lys751Gln polymorphism is one of the most extensively studied genetic markers within the ERCC2 gene. The substitution of lysine for glutamine can significantly affect the interaction between ERCC2 protein and its helicase activator, proteasome 44. Consequently, this obstruction may hinder DNA damage repair mechanisms and increase susceptibility to tumorigenesis (36). Moreover, variations in the ERCC2 gene not only impact tumor susceptibility but also affect sensitivity to platinum-based chemotherapeutics. Platinum-based drugs are commonly utilized as first-line treatments for gynecological tumors; however, platinum resistance presents a pressing challenge that necessitates resolution.

The NER pathway can repair cross-links formed between platinum drugs such as cisplatin, carboplatin, and oxaliplatin with guanine. Consequently, the mRNA and protein expression levels of components involved in the NER pathway will affect the efficacy and toxicity of cisplatin. Studies have shown that ERCC2 expression increases in glioma and colon cancer cells resistant to cisplatin (37). However, platinum-based chemotherapy in ovarian cancer patients with the ERCC2 Lys751Gln polymorphism was associated with a reduced risk of death, particularly among those with heterozygous genotypes (38). Further research is needed to elucidate the relationship between ERCC2 Lys751Gln polymorphism and tumor susceptibility, especially concerning gynecological cancers.

Numerous studies have investigated the relationship between ERCC2 gene polymorphisms and gynecological tumors; however, their findings remain controversial. Shao et al. (39) and Li et al. (40) respectively reported that the ERCC2 Asp312Asn polymorphism may increase the risk of cervical cancer and ovarian cancer. In contrast to these findings, Zhang et al. (41) indicated no association between the Lys751Gln polymorphism and ovarian cancer risk. Furthermore, Tian et al. (42) also found no correlation between the ERCC2 Lys751Gln and Arg156Arg polymorphisms and ovarian cancer risk. This may be related to the small sample size and clinical heterogeneity of the studies. Therefore, we conducted this more systematic and comprehensive study to obtain more reliable results.

Compared to previous studies, this study includes a larger sample size. We found that the ERCC2 Lys751Gln polymorphism was significantly associated with ovarian cancer, while the ERCC2 Asp312Asn and Arg156Arg polymorphisms did not exhibit any relationship with susceptibility to ovarian cancer. These findings align with the conclusions drawn by Li et al. (40) and Tian et al. (42). However, in contrast to the results reported by Shao et al. (39), our study indicated no significant correlation between the ERCC2 Lys751Gln polymorphism and susceptibility to cervical cancer. Notably, Shao et al.’s study included only three studies (comprising 480 cases and 577 controls), whereas our analysis encompassed a larger population (745 cases and 844 controls), thereby enhancing the reliability of our results. In a study investigating the relationship between ERCC2 polymorphisms and mRNA levels using Polymerase Chain Reaction (PCR) technology, it was observed that the Lys751Gln polymorphism has the potential to influence local folding and mRNA stability. This alteration may lead to changes in the secondary structure of the encoded mRNA, thereby impacting its biological functions. Conversely, the Asp312Asn polymorphism merely reduces mRNA levels without inducing structural modifications, which consequently does not affect its biological functions (43). This distinction may elucidate why the Lys751Gln polymorphism is associated with increased susceptibility to gynecological tumors, whereas the Asp312Asn polymorphism does not exhibit such an association.

Furthermore, during subgroup analyses based on race, we discovered that individuals carrying the Lys751Gln polymorphism exhibited an elevated risk for developing gynecological tumors within both Caucasian and African populations. Additionally, subgroup analysis by population found that in the hospital population, the Lys751Gln polymorphism was associated with susceptibility to gynecological tumors. However, the ERCC2 Asp312Asn polymorphism may reduce the susceptibility to cervical cancer in Asian women.

At present, an experiment conducted in Japan has demonstrated that the ERCC2 Lys751Gln polymorphism is associated with an increased risk of lung cancer. Furthermore, the combination of multiple high-risk genotypes—including CYP1A1 rs4646903, GSTM1 deletion polymorphism, and ERCC2 Lys751Gln—elevates the risk of lung cancer by a factor of 5.94 (44). Additionally, ERCC2 Lys751Gln and Asp312Asn polymorphisms are prognostic factors for locally advanced head and neck cancer after definitive cisplatin chemoradiotherapy. The ERCC2 gene Lys751Gln and Asp312Asn SNP have been shown to correlate with a diminished response to cisplatin-based chemotherapy or radiotherapy in patients with head and neck cancer (45). Looking ahead, there is potential for using the polymorphisms of the ERCC2 gene as biomarkers in oncology. For instance, these markers could facilitate the identification of high-risk tumor groups and aid in predicting the efficacy of platinum-based radiotherapy and chemotherapy treatments.

Limitations of this study: 1. The included study populations are limited. The majority of included populations are Caucasians, with few studies on Asian and African populations; 2. Sample sizes are insufficient, and the numbers for several subgroup analyses are still very limited; 3. Some included studies do not conform to Hardy-Weinberg equilibrium (HWE), but we conducted subgroup analyses based on these studies; 4. The estimated results from included studies are not adjusted. Most studies did not consider important confounding factors such as gene-gene and gene-environment interactions, age, menopausal status;5. Some studies did not clearly provide information on pathological types, so subgroup analysis was not conducted based on pathological types; 6. The quality scores of some included studies were relatively low, but to conduct a more comprehensive statistical analysis, low-quality literature was not excluded.

In summary, the ERCC2 Lys751Gln polymorphism is associated with an increased risk of gynecological tumors, particularly ovarian cancer. In contrast, the Asp312Asn and Arg156Arg polymorphisms do not appear to elevate susceptibility to gynecological tumors. In addition, the recessive gene model of ERCC2 Asp312Asn may reduce the susceptibility to cervical cancer in the Asian population.

## Data Availability

The original contributions presented in the study are included in the article/supplementary material. Further inquiries can be directed to the corresponding author.

## References

[1] Bray F Laversanne M Sung H Ferlay J Siegel RL Soerjomataram I . Global cancer statistics 2022: GLOBOCAN estimates of incidence and mortality worldwide for 36 cancers in 185 countries. CA Cancer J Clin. (2024) 74:229–63. doi: 10.3322/caac.21834, PMID: , PMID: 38572751

[2] Joo J Yoon KA Hayashi T Kong SY Shin HJ Park B . Nucleotide excision repair gene ERCC2 and ERCC5 variants increase risk of uterine cervical cancer. Cancer Res Treat. (2016) 48:708–14. doi: 10.4143/crt.2015.098, PMID: , PMID: 26130668 PMC4843739

[3] Ferrall L Lin KY Roden RBS Hung CF Wu TC . Cervical cancer immunotherapy: facts and hopes. Clin Cancer Res. (2021) 27:4953–73. doi: 10.1158/1078-0432.CCR-20-2833, PMID: , PMID: 33888488 PMC8448896

[4] Siegel RL Miller KD Wagle NS Jemal A . Cancer statistics, 2023. CA Cancer J Clin. (2023) 73:17–48. doi: 10.3322/caac.21763, PMID: , PMID: 36633525

[5] Hecht JL Mutter GL . Molecular and pathologic aspects of endometrial carcinogenesis. J Clin Oncol. (2006) 24:4783–91. doi: 10.1200/JCO.2006.06.7173, PMID: , PMID: 17028294

[6] Michalska MM Samulak D Jabłoński F Romanowicz H Smolarz B . The R156R ERCC2 polymorphism as a risk factor of endometrial cancer. Tumour Biol. (2016) 37:2171–6. doi: 10.1007/s13277-015-4040-8, PMID: , PMID: 26349749

[7] Khanlarkhani N Azizi E Amidi F Khodarahmian M Salehi E Pazhohan A . Metabolic risk factors of ovarian cancer: a review. JBRA Assist Reprod. (2022) 26:335–47. doi: 10.5935/1518-0557.20210067, PMID: , PMID: 34751020 PMC9118962

[8] Panagopoulou M Panou T Gkountakos A Tarapatzi G Karaglani M Tsamardinos I . BRCA1 & BRCA2 methylation as a prognostic and predictive biomarker in cancer: Implementation in liquid biopsy in the era of precision medicine. Clin Epigenet. (2024) 16:178. doi: 10.1186/s13148-024-01787-8, PMID: , PMID: 39643918 PMC11622545

[9] Chatterjee N Walker GC . Mechanisms of DNA damage, repair, and mutagenesis. Environ Mol Mutagen. (2017) 58:235–63. doi: 10.1002/em.22087, PMID: , PMID: 28485537 PMC5474181

[10] Goode EL Ulrich CM Potter JD . Polymorphisms in DNA repair genes and associations with cancer risk. Cancer Epidemiol Biomarkers Prev. (2002) 11:1513–30. 12496039

[11] Hoag A Duan M Mao P . The role of Transcription Factor IIH complex in nucleotide excision repair. Environ Mol Mutagen. (2024) 65 Suppl 1:72–81. doi: 10.1002/em.22568, PMID: , PMID: 37545038 PMC10903506

[12] Thakur M Muniyappa K . Global genome and transcription-coupled nucleotide excision repair pathway in prokaryotes. J Biosci. (2023) 48:56. doi: 10.1007/s12038-023-00378-8, PMID: , PMID: 38088378

[13] Benhamou S Sarasin A . ERCC2/XPD gene polymorphisms and cancer risk. Mutagenesis. (2002) 17:463–9. doi: 10.1093/mutage/17.6.463, PMID: , PMID: 12435843

[14] Zachayus A Loup-Forest J Cura V Poterszman A . Nucleotide excision repair: insights into canonical and emerging functions of the transcription/DNA repair factor TFIIH. Genes (Basel). (2025) 16:231. doi: 10.3390/genes16020231, PMID: , PMID: 40004560 PMC11855273

[15] Li M Chen R Ji B Fan C Wang G Yue C . Contribution of XPD and XPF polymorphisms to susceptibility of non-small cell lung cancer in high-altitude areas. Public Health Genomics. (2021) 24:189–98. doi: 10.1159/000512641, PMID: , PMID: 33827099

[16] Zhou Q Fu Y Wen L Deng Y Chen J Liu K . XPD polymorphisms and risk of hepatocellular carcinoma and gastric cancer: A meta-analysis. Technol Cancer Res Treat. (2021) 20:1533033821990046. doi: 10.1177/1533033821990046, PMID: , PMID: 33517857 PMC7871355

[17] Wang Y Zhou Y Wang Y Peng C Gao M . Cloning of the XPD gene and its function in Malignant melanoma cells. Oncol Lett. (2020) 20:1803–9. doi: 10.3892/ol.2020.11708, PMID: , PMID: 32724423 PMC7377112

[18] Bajpai D Banerjee A Pathak S Thakur B Jain SK Singh N . Single nucleotide polymorphisms in the DNA repair genes in HPV-positive cervical cancer. Eur J Cancer Prev. (2016) 25:224–31. doi: 10.1097/CEJ.0000000000000159, PMID: , PMID: 25812040

[19] Bernard-Gallon D Bosviel R Delort L Fontana L Chamoux A Rabiau N . DNA repair gene ERCC2 polymorphisms and associations with breast and ovarian cancer risk. Mol Cancer. (2008) 7:36. doi: 10.1186/1476-4598-7-36, PMID: , PMID: 18454848 PMC2394522

[20] Cincin ZB Iyibozkurt AC Kuran SB Cakmakoglu B . DNA repair gene variants in endometrial carcinoma. Med Oncol. (2012) 29:2949–54. doi: 10.1007/s12032-012-0162-7, PMID: , PMID: 22271435

[21] Costa S Pinto D Pereira D Vasconcelos A Afonso-Lopes C Osório T . Importance of xeroderma pigmentosum group D polymorphisms in susceptibility to ovarian cancer. Cancer Lett. (2007) 246:324–30. doi: 10.1016/j.canlet.2006.03.014, PMID: , PMID: 16677755

[22] Datkhile KD Durgawale PP Patil MN Gudur RA Gudur AK Patil SR . Impact of polymorphism in base excision repair and nucleotide excision repair genes and risk of cervical cancer: A case-control study. Asian Pac J Cancer Prev. (2022) 23:1291–300. doi: 10.31557/APJCP.2022.23.4.1291, PMID: , PMID: 35485688 PMC9375594

[23] Doherty JA Weiss NS Fish S Fan W Loomis MM Sakoda LC . Polymorphisms in nucleotide excision repair genes and endometrial cancer risk. Cancer Epidemiol Biomarkers Prev. (2011) 70:1873–82. doi: 10.1158/1055-9965.EPI-11-0119, PMID: , PMID: 21750170 PMC3169742

[24] He X Ye F Zhang J Cheng Q Shen J Chen H . Susceptibility of XRCC3, XPD, and XPG genetic variants to cervical carcinoma. Pathobiology. (2008) 75:356–63. doi: 10.1159/000164220, PMID: , PMID: 19096231

[25] Jakubowska A Gronwald J Menkiszak J Górski B Huzarski T Byrski T . BRCA1-associated breast and ovarian cancer risks in Poland: no association with commonly studied polymorphisms. Breast Cancer Res Treat. (2010) 119:201–11. doi: 10.1007/s10549-009-0390-5, PMID: , PMID: 19360465

[26] Khokhrin DV Khrunin AV Moiseev AA Gorbunov VA Limborskaia SA . Association of polymorphisms in glutathione-S-transferase and DNA repair genes with ovarian cancer risk in the Russian population. Russ J Genet. (2012) 764–6. doi: 10.1134/S1022795412050110, PMID: , PMID: 22988779

[27] Michalska MM Samulak D Romanowicz H Sobkowski M Smolarz B . An association between single nucleotide polymorphisms of lys751GlnERCC2Gene and ovarian cancer in polish women. Adv Med. (2015) 2015:1–6. doi: 10.1155/2015/109593, PMID: , PMID: 26526682 PMC4615857

[28] Mohamed FZ Hussien YM AlBakry MM Mohamed RH Said NM . Role of DNA repair and cell cycle control genes in ovarian cancer susceptibility. Mol Biol Rep. (2013) 40:3757–68. doi: 10.1007/s11033-012-2452-8, PMID: , PMID: 23277402

[29] Monteiro MS Vilas Boas DB Gigliotti CB Salvadori DM . Association among XRCC1, XRCC3, and BLHX gene polymorphisms and chromosome instability in lymphocytes from patients with endometriosis and ovarian cancer. Genet Mol Res. (2014) 13:636–48. doi: 10.4238/2014.January.28.9, PMID: , PMID: 24615029

[30] Romanowicz H Michalska MM Samulak D Malinowski J Szaflik T Bienkiewicz J . Association of R156R single nucleotide polymorphism of the ERCC2 gene with the susceptibility to ovarian cancer. Eur J Obstet Gynecol Reprod Biol. (2017) 208:36–40. doi: 10.1016/j.ejogrb.2016.11.012, PMID: , PMID: 27888704

[31] Smolarz B Michalska MM Samulak D Wójcik L Romanowicz H . Studies of Correlations Between Single Nucleotide Polymorphisms of DNA Repair Genes and Endometrial Cancer in Polish Women. Anticancer Res. (2018) 38(9):5223–9. doi: 10.21873/anticanres.12846, PMID: , PMID: 30194171

[32] Sobczuk A Poplawski T Blasiak J . Polymorphisms of DNA repair genes in endometrial cancer. Pathol Oncol Res. (2012) 18:1015–20. doi: 10.1007/s12253-012-9537-5, PMID: , PMID: 22544315 PMC3448050

[33] Weiss JM Weiss NS Ulrich CM Doherty JA Voigt LF Chen C . Interindividual variation in nucleotide excision repair genes and risk of endometrial cancer. Cancer Epidemiol Biomarkers Prev. (2005) 14(11 Pt 1):2524–30. doi: 10.1158/1055-9965.EPI-05-0414, PMID: , PMID: 16284373

[34] Zhang L Ruan Z Hong Q Gong X Hu Z Huang Y . Single nucleotide polymorphisms in DNA repair genes and risk of cervical cancer: A case-control study. Oncol Lett. (2012) 3:351–62. doi: 10.3892/ol.2011.463, PMID: , PMID: 22740911 PMC3362506

[35] Zhao Z Zhang A Zhao Y Xiang J Yu D Liang Z . The association of polymorphisms in nucleotide excision repair genes with ovarian cancer susceptibility. Biosci Rep. (2018) 38. doi: 10.1042/BSR20180114, PMID: , PMID: 29669843 PMC6013708

[36] Ding DP Ma WL He XF Zhang Y . XPD Lys751Gln polymorphism and esophageal cancer susceptibility: a meta-analysis of case-control studies. Mol Biol Rep. (2012) 39:2533–40. doi: 10.1007/s11033-011-1005-x, PMID: , PMID: 21667112

[37] Bowden NA . Nucleotide excision repair: why is it not used to predict response to platinum-based chemotherapy? Cancer Lett. (2014) 346:163–71. doi: 10.1016/j.canlet.2014.01.005, PMID: , PMID: 24462818

[38] Khrunin AV Moisseev A Gorbunova V Limborska S . Genetic polymorphisms and the efficacy and toxicity of cisplatin-based chemotherapy in ovarian cancer patients. Pharmacogenom J. (2010) 10:54–61. doi: 10.1038/tpj.2009.45, PMID: , PMID: 19786980

[39] Shao X Yang X Liu Y Song Q Pan X Chen W . Genetic polymorphisms in DNA repair genes and their association with risk of cervical cancer: A systematic review and meta-analysis. J Obstet Gynaecol Res. (2022) 48:2405–18. doi: 10.1111/jog.15325, PMID: , PMID: 35732591

[40] Li J Pan L Qin X Chu H Mu H Wan G . Association between ERCC2 rs13181 polymorphism and ovarian cancer risk: an updated meta-analysis with 4024 subjects. Arch Gynecol Obstet. (2017) 296:551–8. doi: 10.1007/s00404-017-4443-4, PMID: , PMID: 28676967

[41] Zhang W Zhang Z . Associations between XRCC2 rs3218536 and ERCC2 rs13181 polymorphisms and ovarian cancer. Oncotarget. (2016) 7:86621–9. doi: 10.18632/oncotarget.13361, PMID: , PMID: 27863412 PMC5349940

[42] Tian Y Lin X Yang F Zhao J Yao K Bian C . Contribution of xeroderma pigmentosum complementation group D gene polymorphisms in breast and ovarian cancer susceptibility: A protocol for systematic review and meta analysis. Med (Baltimore). (2020) 99:e20299. doi: 10.1097/MD.0000000000020299, PMID: , PMID: 32481313 PMC7249878

[43] Wolfe KJ Wickliffe JK Hill CE Paolini M Ammenheuser MM Abdel-Rahman SZ . Single nucleotide polymorphisms of the DNA repair gene XPD/ERCC2 alter mRNA expression. Pharmacogenet Genomics. (2007) 17:897–905. doi: 10.1097/FPC.0b013e3280115e63, PMID: , PMID: 18075460

[44] Kiyohara C Horiuchi T Takayama K Nakanishi Y . Genetic polymorphisms involved in carcinogen metabolism and DNA repair and lung cancer risk in a Japanese population. J Thorac Oncol. (2012) 7:954–62. doi: 10.1097/JTO.0b013e31824de30f, PMID: , PMID: 22525558

[45] Guberina M Sak A Pottgen C Tinhofer-Keilholz I Budach V Balermpas P . ERCC2 gene single-nucleotide polymorphism as a prognostic factor for locally advanced head and neck carcinomas after definitive cisplatin- based radiochemotherapy. Pharmacogenom J. (2021) 21:37–46. doi: 10.1038/s41397-020-0174-1, PMID: , PMID: 32546699 PMC7840506

