# Correction: Association between ERCC2 Lys751Gln, Asp312Asn, and Arg156Arg polymorphisms and gynecological cancer susceptibility: a meta-analysis

**DOI:** 10.3389/fonc.2025.1702750

**Published:** 2025-09-25

**Authors:** Fen Chen, Jiayang Yu, Chun-Guang Wang

**Affiliations:** Department of Oncology, Yongchuan Hospital of Chongqing Medical University, Chongqing, China

**Keywords:** gynecological neoplasms, ovarian cancer, cervical cancer, endometrial cancer, polymorphism, meta-analysis, ERCC2

Authors “Fen Chen and Chun-Guang Wang” were erroneously omitted as equal contributing author.

The correct author list reads:

“Fen Chen#, Jiayang Yu# and Chun-Guang Wang*”.

“#These authors have contributed equally to this work and share first authorship”

The original version of this article has been updated.

